# Supplementary material for: Chromosome Distribution of Highly Conserved Tandemly Arranged Repetitive DNAs in the Siberian Sturgeon (Acipenser baerii)
Source: Genes (Basel). 2020 Nov 20;11(11):1375. doi: 10.3390/genes11111375 (PMC7699875; doi:10.3390/genes11111375)
Supplement: Supplementary file 1 [file genes-11-01375-s001.pdf]

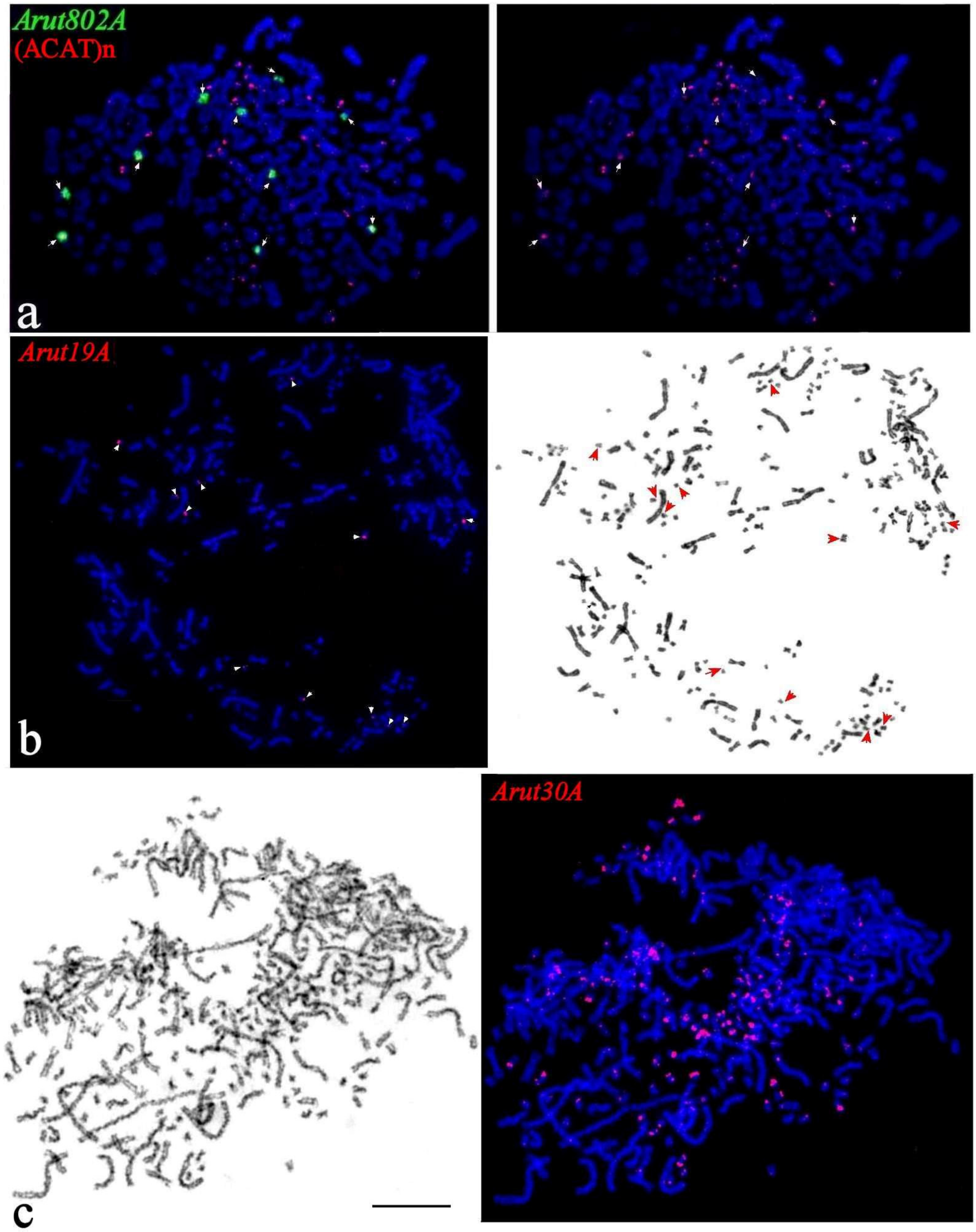

Figure S1. FISH (Fluorescence in situ hybridization) of repetitive DNA probes on metaphase plates of the Siberian sturgeon. **a** Satellites $(ACAT)_n$ (red) and *Arut802A* (green), on the right the same plate with $(ACAT)_n$ only, arrows mark chromosomes with co-localized probes; **b** satellite *Arut19A* (red), on the right DAPI inverted plate, arrows mark chromosomes with signals; **c** satellite *Arut30A* (red) on GTG (G-banding by trypsin using Giemsa) differential stained metaphase plate (left). Scale bar is 10 μm.

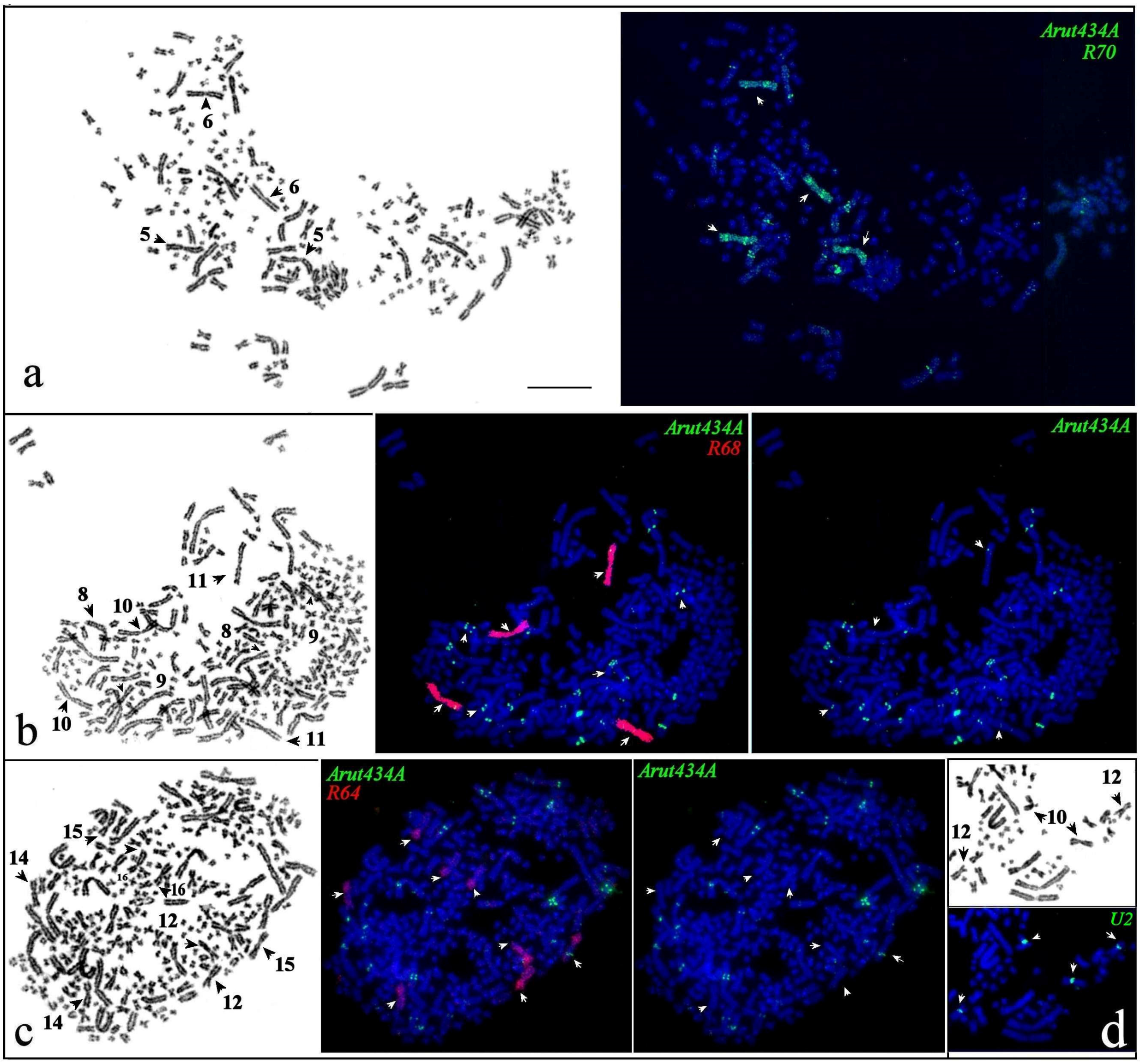

Figure S2. FISH of repetitive and microdissection-derived painting probes (right) on GTG-banded metaphase plates (left) of the Siberian sturgeon (**a**–**c**). **a** *Arut434A* (green) and painting probe for sterlet chromosome 3 (R70, green) co-localized on sturgeon chromosomes 5 and 6; **b** *Arut434A* (green) and painting probe for sterlet chromosome 6 (R68, red); **c** *Arut434A* (green) and painting probe for chromosome ARUT8 (R64, red); **d** *Arut802* (red) and U2 snRNA probe (green) on a part of metaphase plate of sterlet. Chromosomes with signals are marked and numbered. Scale bar is 10 µm.

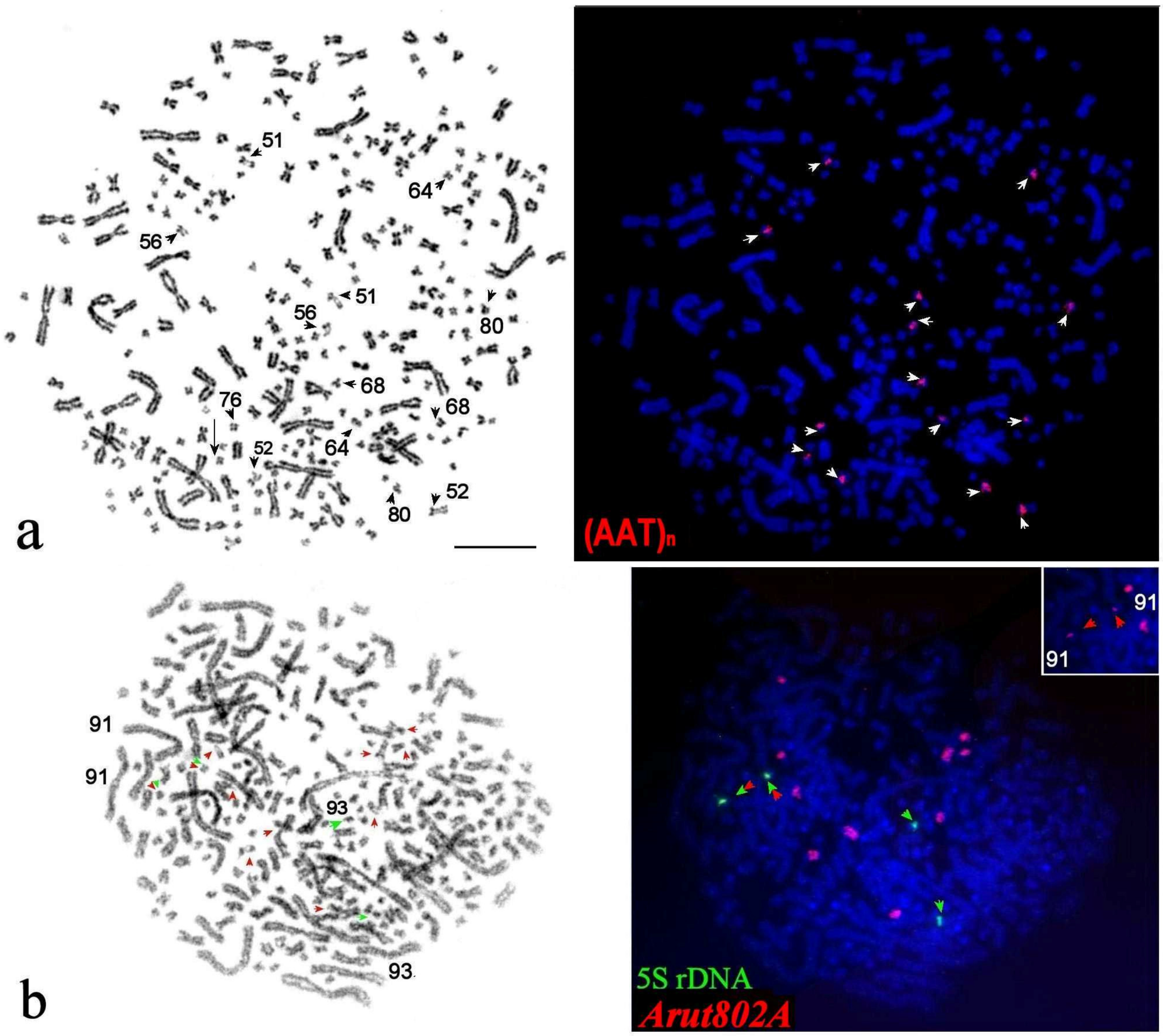

Figure S3. FISH of repetitive probes (right) on GTG-banded metaphase plates (left) of the Siberian sturgeon (**a, b**). **a** $(AAT)_n$ (red); **b** 5S rDNA (green) and *Arut802A* (red), in the right corner is an inset part of the metaphase with overlapped signals. Chromosomes with signals are marked and numbered, the double arrows indicate chromosomes with co-localized probes. Scale bar is 10 μm.

Table S1. Chromosome localization of sterlet-derived chromosome-specific probes on orthologs of *Acipenser ruthenus* (ARUT) and *Acipenser baerii* (ABAE)

#	Molecular markers		Chromosome numbers		Characteristics
	tandem repeats	ARUT-derived chromosome specific probe	ARUT	ABAE	
1	*Arut 434A*	1p	1	1	- ABAE 1 and 2: strong signals as on ARUT 1;<br>- ABAE 1: a single signal,<br>ABAE 2: two signals
				2	
		2p	2	3	- ABAE 3 and 4: weak signals as on ARUT 2;<br>- ABAE 4: weak single signal;<br>- ABAE 3: double weak signals
				4	
2	*Arut 434A*	3	3	5	- ABAE 5 and 6: signals on both arms
				6	
		4	4	7	- one of ARUT 4 ohnologs underwent fission resulted in two acrocentrics;<br>- ABAE 102: strong signals
				101	
				102	

3	*Arut 434A*	5	5	8	- ABAE 8 and 9: multiple signals on q-arms;<br><br>- ABAE 9 the distance between the blocks is less than on ABAE 8
				9	
		6	6	10	
				11	
4	*Arut 434A*	7, 14	7	13	- one of the orthologs of ARUT 7 divided into 2 acrocentrics:<br><br>- ABAE 108;<br><br>- an arm of the submetacentric chromosome ABAE 23;<br><br>- reduced block on on ABAE 23 and 108
				23	
				108	
			14	103	- distal end of ABAE 103;<br><br>- interstitial block on ABAE 104<br><br>- ABAE 103 is bigger than ABAE 104, ABAE 104 is close in size and morphology to ARUT 14
				104	
5	*Arut 434A*	8	8	12	- ABAE 12 labelled brighter than ABAE 14 by ARUT 8 probe;

				14	- missing signals of ARUT434A
			9	15	- p-arms of the both paralogs produce bright signals by ARUT 8 probe;<br><br>- *Arut434A* repeat marks one homolog of ABAE 15 brighter
				16	
6	U2	10	10cent	19p cent	- ABAE 19 and 20 are similar
				20p cent	
			12cent	25pq cent	- ABAE 25 and 35 are different in size and G-banding patterns
				35pq cent	
7	*28S/18S rDNA*	-	30	30	- ABAE 30 and 50 are different in size and G-banding pattern;<br><br>- ABAE 30 is bigger than ARUT30, and, probably, underwent a fusion with another element;<br><br>- ABAE 50 are heteromorphic: one of homologs is similar to ARUT 30, while the other is smaller due to reduced p-arm
				50	
			-		
8	*28S/18S rDNA,*<br><br>*Arut 802A,*<br><br>*ArutF 26A,*<br><br>*(AAT)n*	-	31	51	- ABAE 51 and 52 are similar to ARUT 31 and 32, respectively<br><br>- ABAE 55 and 56 are smaller than their sterlet orthologs<br><br>- ABAE 63 probably arose after AcR2;
				56	

				63	- the size of *Arut26* and (*AAT)$_n$* blocks on ABAE 51, 52 and 56 are different
			32		
				52	
				55	
9	*Arut F26A,*<br><br>*(AAT)$_n$*	-	39	68	
				76	
			40	64	
				80	
10	*5S rDNA*<br><br>*Arut 219A*	-	41	91	- *5S rDNA* and *Arut219A* colocalized on ABAE91 and 93<br><br>- ABAE91 and 93 are different in morphology and G-banding pattern;<br><br>- *Arut19A, Arut40A* and *Arut57A* colocalized with *Arut219;*<br><br>- *5S rDNA* and *Arut802A* co-localized on ABAE 91
				93	
11	*Arut F167A*	57 (R3)	57	86q	- ABAE 86q and ABAE 100q are orthologs of ARUT 57;<br><br>- fusion with ABAE 86p;<br><br>- fusion with ABAE 100p
				100q	

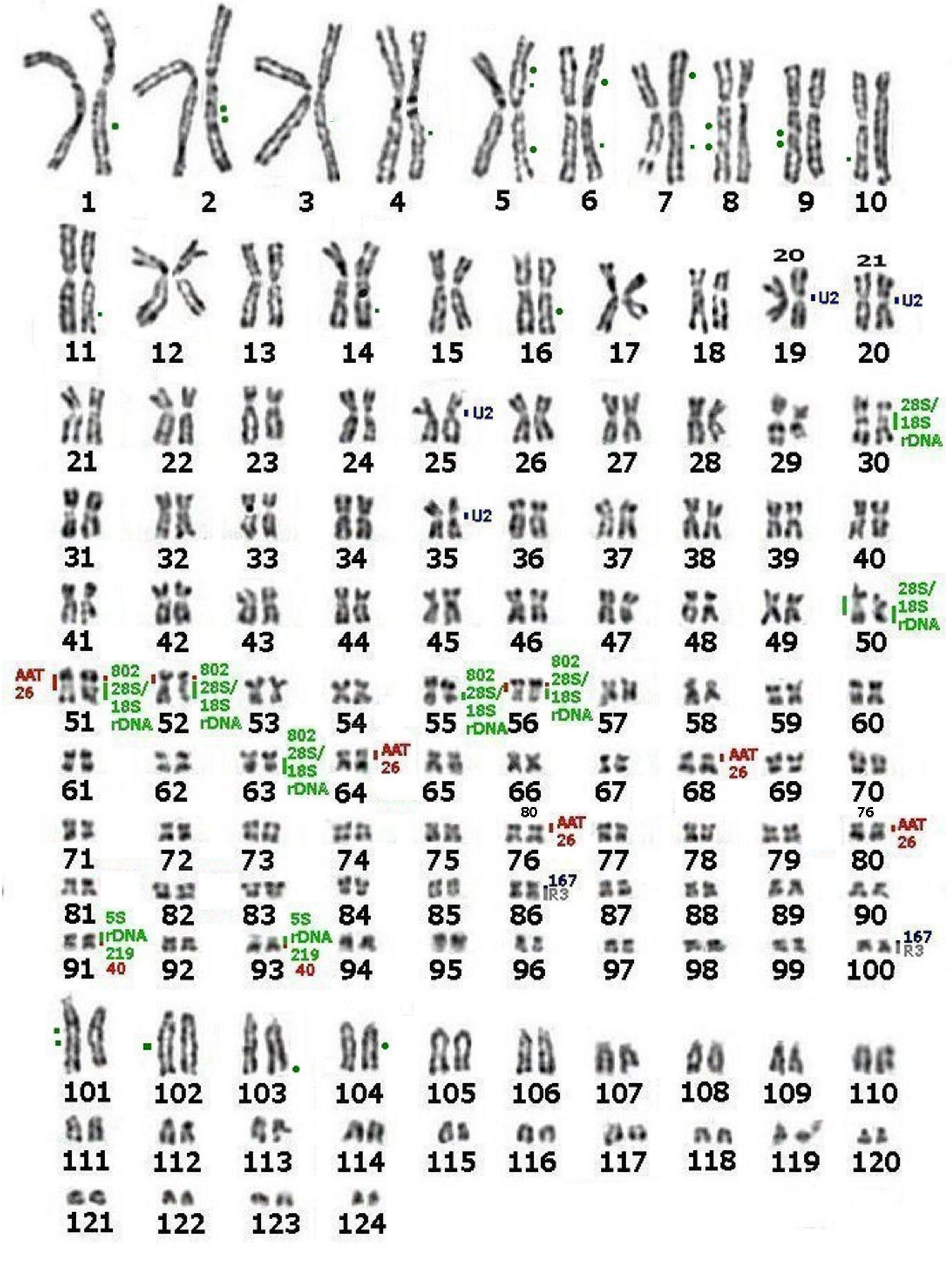

Figure S4. GTG-banded chromosomes Siberian sturgeon with assigned probes: *Arut434A* (434), U2 snRNA genes (U2), ribosomal major cluster (28S/18S rDNA), *Arut26A* (26), *Arut802A* (802), $(AAT)_n$, 5S ribosomal DNA (5S rDNA), *Arut40A* (40), *Arut167A* (167), *Arut219A* (219), chromosome specific probe ARUT 57 (R3).
